# Inhibition Studies of Expansion Damage in Medium–Low Reactivity Limestone by Fly Ash

**DOI:** 10.3390/ma17102422

**Published:** 2024-05-17

**Authors:** Shaocong Dai, Xinyu Zhang, Wei Li, Zhongyang Mao, Xiaojun Huang, Min Deng, Bi Chen

**Affiliations:** 1 College of Material Science and Engineering, Nanjing Tech University, Nanjing 211816, China; 2 College of Emergency Management, Nanjing Tech University, Nanjing 211816, China

**Keywords:** alkali–carbonate reaction, dolomitic limestone, inhibition, cement alkali content, fly ash

## Abstract

Expansion damage in medium–low reactivity dolomite limestone poses significant challenges in construction and engineering projects. This study investigates the potential of fly ash in inhibiting expansion damage in such limestone formations based on RILEM AAR-5 method. Through a series of laboratory experiments, various proportions of fly ash instead of cement, respectively, were prepared and subjected to varying alkali content conditions immersion tests to simulate expansion conditions. The expansion rates and extents were monitored and compared between pure limestone samples and those mixed with different proportions of fly ash. Additionally, scanning electron microscopy (SEM) analysis was employed to investigate the microstructure of the dolomite limestone–fly ash mixtures to understand the inhibition mechanisms. Results indicate that fly ash demonstrates promising inhibitory effects on expansion damage in medium–low reactivity dolomite limestone across the addition of 40% fly ash and alkali content of 0.70%. The reaction products are calcite, brucite, and a mixture of Mg-Si-Al phases and the reaction area is within 100 μm from the boundary when the cement alkali content is 1.50% without any fly ash. However, no reaction products were found at the boundary after adding 40% fly ash when lowering the cement alkali content to 0.70%. This research contributes to a better understanding of the interaction between fly ash and dolomite limestone in inhibiting expansion damage, providing valuable insights for engineering applications.

## 1. Introduction

Alkali–aggregate reaction (AAR) is a phenomenon that may occur in concrete structures, typically referring to the reaction between the alkaline components in concrete and certain aggregates containing reactive minerals (such as siliceous or carbonate rocks) [1]. This reaction can result in expansion and cracking, ultimately leading to damage to the concrete structure. AAR typically falls into two types: alkali–silica reaction (ASR) and alkali–carbonate reaction (ACR). ASR refers to the reaction of alkalis with the siliceous minerals in the aggregate, producing a gel-like material that causes the expansion of concrete volume [2]. ACR involves the reaction of alkalis with carbonate minerals (such as dolomitic rocks), resulting in the formation of alkali metal carbonates, which also leads to volume expansion.

In the early 1950s, Canada and other countries successively discovered severe mesh cracking in concrete using carbonate aggregate. In 1963, Gillott [3] discovered and proposed the ACR, in which in the presence of alkali, dolomite crystals inside dolomite, dolomitic chert, and gray dolomite, which are used as concrete aggregates, react with alkali to form brucite, calcite, and CO_3_^2−^. According to thermodynamic calculations, this reaction process occurs spontaneously. In addition, the hydrated Ca(OH)_2_ in the concrete slurry, which undergoes hydration, continues to react with the CO_3_^2−^ ions in Equation (1) to produce hydroxide ions once again, with the reaction formula shown in Equations (1) and (2):

CaMg(CO_3_)^2^ + 2OH^−^ = Mg(OH)_2_ + CaCO_3_ + CO_3_^2−^ (1)

CO_3_^2−^ + Ca(OH)_2_ = CaCO_3_ + 2OH^−^ (2)

Cody and Deng [4,5] favored the dedolomitization reaction (ADR) to describe ACR. Chen et al. [6,7] illustrated the capability to differentiate between ASR and ACR within carbonate systems using Tetramethylammonium hydroxide (TMAH). They further asserted that the primary culprit behind ACR is the ADR. Li [8] discovered that the dolomitization reaction process primarily involves OH^−^ initially reacting on the surface of dolomite.

**Figure 1:**
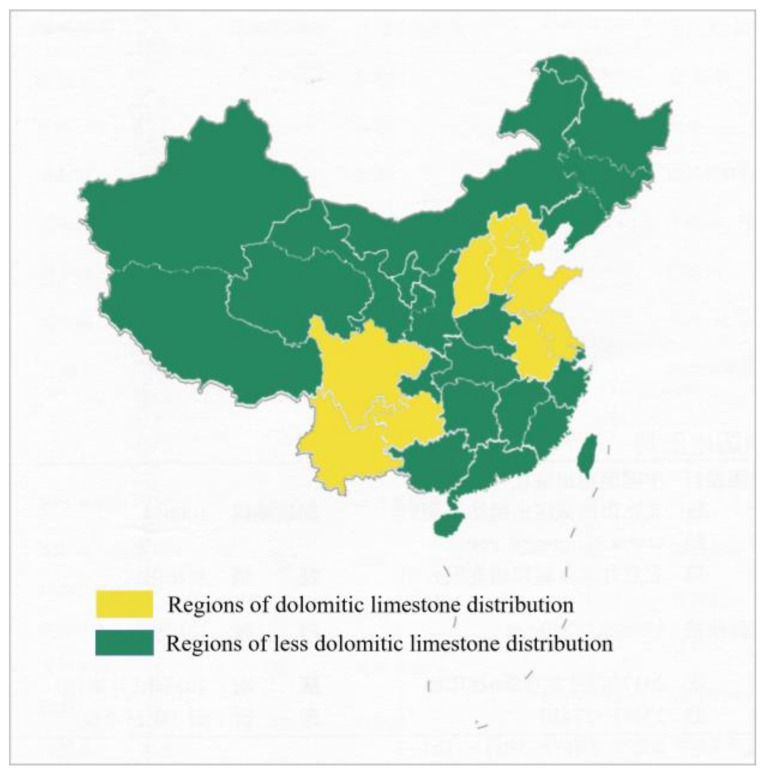
Regions of dolomitic limestone distribution, including Beijing, Tianjin, Hebei, Shanxi, Sichuan, Shandong, Guizhou, Anhui, Jiangsu, Yunnan, and Guizhou.

**Figure 2:**
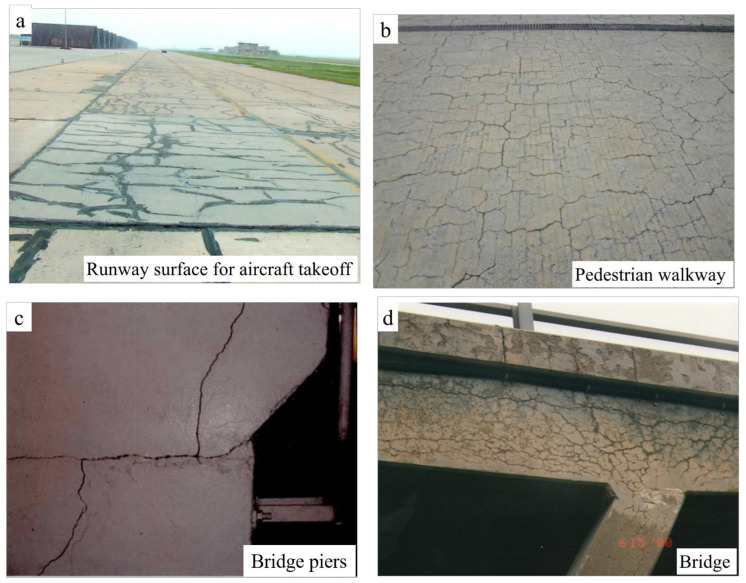
Cases of ACR damage. (**a**) Runway surface for aircraft takeoff in Shandong, China (**b**) Pedestrain walkway in Shandong, China (**c**) Bridge piers in Tianjin, China (**d**) Bridge in Tianjin, China.

Dolomitic rocks are widely distributed in China (as seen in Figure 1) and are the most commonly used rock aggregate in concrete due to their ease of extraction and processing. Since Deng discovered severe cracking damage in concrete structures using carbonate rock aggregates in Shandong Province and Tianjin, China, some other locations have also experienced similar issues, as indicated in Figure 2. Analysis and research identified the cause as ACR. Due to the formation mechanism of carbonate rocks, the limitations of engineering conditions, and economic factors, it is impractical to use entirely non-alkaline reactive aggregates. Identifying aggregate activity and adopting corresponding inhibition measures against alkali–aggregate reaction types are the main methods to prevent damage from alkali–aggregate reactions. This study proposes corresponding inhibition measures and methods to avoid significant economic losses caused by alkali–aggregate reactions in concrete structural engineering, which is of great guiding significance.

To mitigate ACR, numerous researchers both domestically and internationally have conducted extensive investigations into preventive measures. A plethora of experimental studies and engineering practices have demonstrated that the inclusion of fly ash in concrete mixtures not only retards or suppresses ACR but also enhances other concrete properties, conserves resources, and safeguards the environment [9,10,11]. Mineral admixtures such as silica fume, pulverized fly ash and granulated blast furnace slag are widely used to inhibit ACR expansion [12,13,14]. The effectiveness of mineral admixtures in controlling ACR is generally derived from the decreasing of content of cementitious materials, lowering OH^−^ concentrations in pore solutions by the adsorption of low Ca/Si C-S-H gels and resisting the migration of ions in pore solutions of concrete by fining pores [15]. Nevertheless, there has been limited research into ACR inhibition. Deng [5] conducted an assessment of the inhibitory effects of mineral admixtures, sulfoaluminate cement, and persulfate cement on rocks in the Kingston, Canada, utilizing the concrete microbar method. Sulfoaluminate cement, persulfate cement, and low-alkali Portland cement, blended with high dosages of PFA (pulverized fuel ash), BFS (blast furnace slag), or SF (silica fume), effectively prevented concrete expansion and cracking induced by alkali–dolomite reaction. In cases where Portland cement with an equivalent alkali content of 0.43% and reactive dolomitic limestone from Kingston, Canada, was employed, it became imperative to substitute the cement with 70% PFA, 90% BFS, or 30% SF. The essence of the issue lies in significantly lowering the pH of the concrete pore solution. Shehata [16] assessed the prolonged impacts of supplementary cementitious materials (SCMs) on ACR over a span of up to 10 years utilizing a concrete prism test. While certain types of SCMs exhibited greater effectiveness in diminishing expansion, none proved efficacious in curbing it over the long haul. Blending 10% reactive aggregate with 90% non-reactive aggregate proved effective in attaining the threshold of achieving expansion below 0.040% at the end of the first year. However, the expansion of the specimen escalated to 0.074% after a decade of placement at room temperature. Joshaghani [17] evaluated the effectiveness of ACR inhibition in both short and long terms using two types of admixtures, namely trass and fly ash. The experiments revealed that trass was not highly effective in inhibiting ACR compared to fly ash. Ren [18] conducted studies using the concrete microbar method and concrete prism method to investigate the impact of mineral admixtures on ACR expansion in dolomitic rocks. They found that 30% fly ash could temporarily inhibit alkali carbonate reaction, with the primary mechanism being the refinement of pore structure and reduction in the migration rate of Na^+^ and K^+^ ions into the pore solution.

In published papers, although there are many reports related to AAR inhibition by fly ash, this is mostly for ASR, with very few reports for ACR. There is still no definitive answer as to whether the use of fly ash has an inhibitory effect on ACR, and many of the inhibition methods are currently based on a modification of the alkali activity test method (RILEM AAR-5; RILEM AAR-2), which is based on a conditioning environment of 80 °C, 1 mol/L NaOH solution with a cement alkali content of 1.50%. This will have an accelerating effect in the experiment, but due to the harshness of the curing environment in the past, it could not be suppressed even with the use of fly ash, and cements with such a high alkali content would not be used in practical engineering applications.

The objective of this study was to investigate the effect of fly ash on the inhibition of alkali carbonate reaction (ACR) in low and moderately reactive dolomitic limestones and to assess the suitable conditions for the application of fly ash in these two types of dolomitic limestones by using different alkaline environments. In this study, the efficacy of fly ash in inhibiting the alkali carbonate reaction (ACR) swelling of dolomitic limestone in different alkaline environments was evaluated by adjusting the admixture ratio of fly ash using the concrete micro-column test method based on the RILEM AAR-5 standard. Scanning electron microscopy (SEM) was utilized to observe the morphology of dolomite limestone after the reaction in order to observe the inhibition effect of fly ash on dolomite limestone, and then application guidelines are proposed for the specific conditions of dolomite limestone.

## 2. Materials and Methods

### 2.1. Materials

#### 2.1.1. Cement

**Figure 3:**
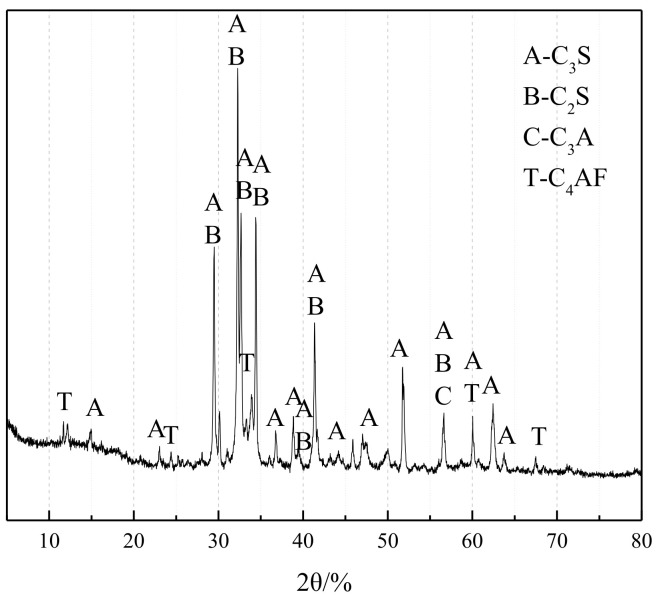
XRD pattern of P•II 52.5 cement.

The cement used in the experiment was P•II 52.5 cement produced by Nanjing Jiangnan Onoda Co., Ltd., Nanjing, China with an alkali content of (Na_2_O_eq_ = Na_2_O + 0.658K_2_O) of 0.54%. The chemical composition is shown in Table 1, and the XRD is shown in Figure 3.

#### 2.1.2. Fly Ash

**Figure 4:**
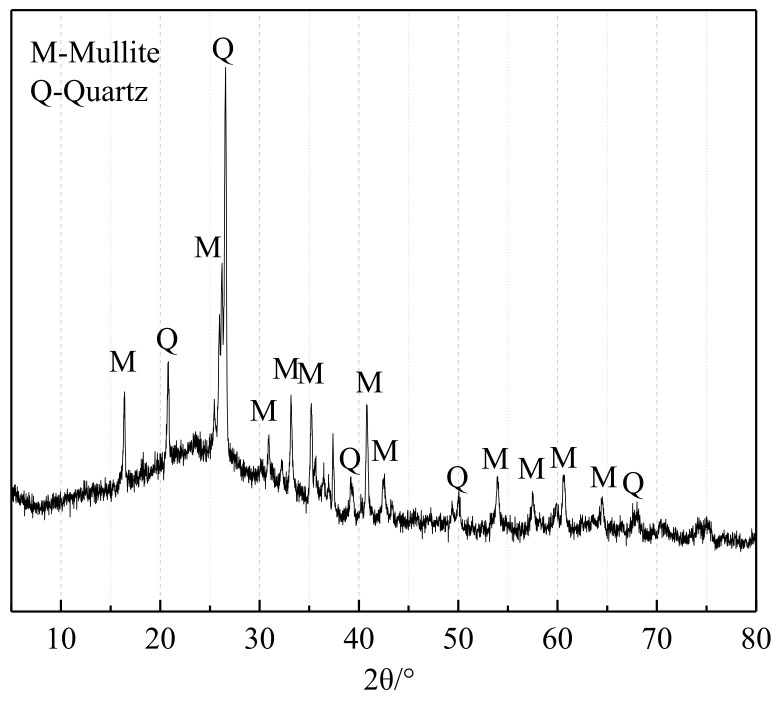
XRD pattern of FA.

The fly ash (FA) used in the experiment is Class II, which complies with the GB/T 1596-2017 standard [19], produced by Pudi Mixing Station in Nanjing, China. Its density is 2.24 g/cm^3^. Its chemical composition is shown in Table 2. Figure 4 shows the XRD pattern of FA, wherein the primary components identified are mullite and quartz.

#### 2.1.3. Aggregates

**Figure 5:**
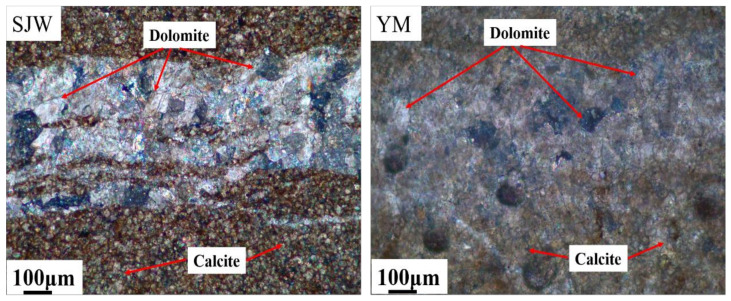
Petrographic micrographs of dolomitic rocks SJW and YM.

**Figure 6:**
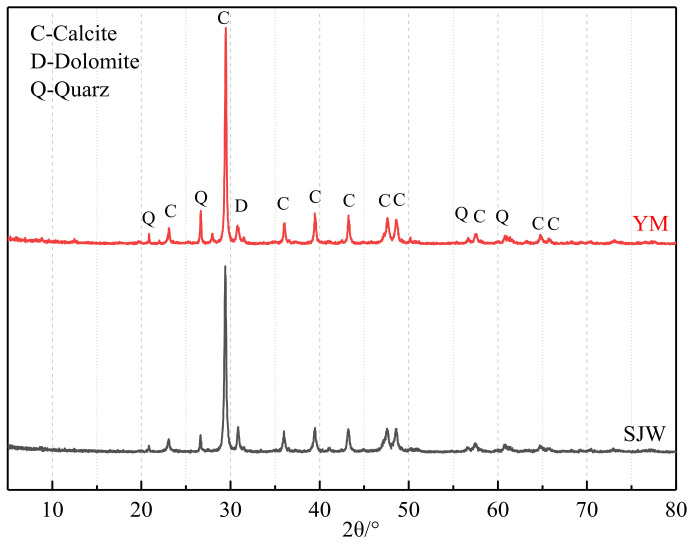
XRD patterns of rocks SJW and YM.

The dolomitic rocks selected for this experiment were sourced from the dolomitic limestone designated as SJW and YM, located in the quarry of Sansui County, Guizhou. The experiment used aggregate with a particle size of 5–10 mm, with a single gradation. Figure 5 shows the petrographic map of SJW and YM, revealing a mosaic-type structure in the dolomite of these rocks. From the chemical composition table provided in Table 3 and the XRD pattern depicted in Figure 6, it is evident that the primary components of both rocks are calcite and dolomite.

### 2.2. Experimental Methods

#### 2.2.1. Inhibition Experiments with Simulated Pore Solutions

**Figure 7:**
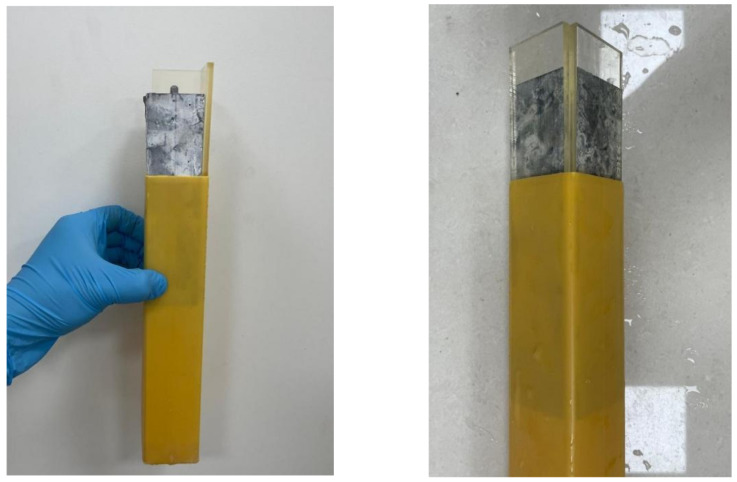
Curing of concrete microbar specimens.

In this study, the ultra-accelerated mortar bar method (RILEM AAR-2) [20] was employed to investigate the potential alkali–silica reactivity of SJW and YM rock samples, while rapid preliminary screening test for the carbonate aggregate method (RILEM AAR-5) [21] was utilized to assess their alkali–carbonate reactivity. Subsequently, based on the RILEM AAR-5 concrete microbar method, concrete microbars with dimensions of 40 mm × 40 mm × 160 mm were prepared. The aggregate mass was 900 g, and the cement-to-aggregate mass ratio was 1:1, with an aggregate particle size ranging from 5 mm to 10 mm. P•II 52.5 silicate cement with an alkali content of 0.54% was used. The alkali content of the cement was adjusted to 1.50 wt% and 0.70 wt% using analytically pure NaOH reagent. Additionally, 0, 20%, 30%, and 40% of fly ash were substituted for an equal mass of cement, with a water-to-binder ratio of 0.32. After molding for 24 h and demolding, the initial length L_0_ of the specimens was measured using a length comparator. Subsequently, the concrete microbars with different alkali contents were placed in 80 °C solutions of 60 mL 1.5 mol/L and 60 mL 0.7 mol/L NaOH for curing. To minimize the influence of external curing solutions on the microbars, they were placed in boxes measuring 45 mm × 45 mm × 180 mm and filled with two 5 mm × 40 mm × 160 mm acrylic plates to fill the gaps, the model is as shown in Figure 7. After a specific curing period, the concrete microbar specimens were retrieved and their lengths, L_D_, were accurately measured after cooling. Through this series of experiments, the aim was to elucidate the reaction activity of SJW and YM rock samples under different alkali concentration environments.

The formula for calculation is as follows:

Pt = (L_0_ − 2b)/(L_t_ − L_0_) × 100%

where:

Pt is the expansion rate after t days of curing, in %;

L_t_ is the test piece length after t days of curing, in mm;

L_0_ is the initial length of the test piece, in mm;

b is the length of the nail embedded in the concrete, in mm.

#### 2.2.2. Micro-Structure Analysis

**Figure 8:**
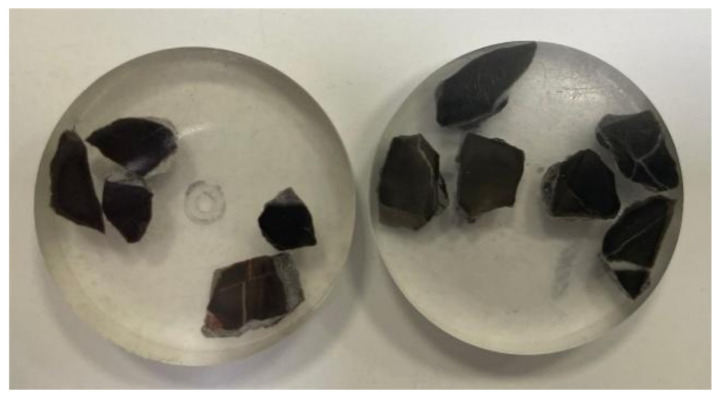
Photomicrograph of SJW sample production.

To investigate the microstructural changes in concrete microbars after 120 days of reaction, SEM samples need to be prepared. Selected samples of SJW with a cement alkali content of 1.50%, maintained at 80 °C, SJW with a cement alkali content of 1.50% doped with 40% FA, and SJW with a cement alkali content of 0.70% doped with 40% FA were selected for the study. Firstly, the concrete microbars were gently crushed, and the rock particles were carefully extracted. Next, the reacted rock particles were placed into a mold and filled with epoxy resin for curing. Then, precise cutting was performed until the rock surface was fully exposed. Subsequently, the surfaces of the rock samples were finely polished and made into slides using the AutoMet 250 grinder–polisher produced by Buehler, Lake Bluff, IL, USA. After ensuring a smooth and traceless surface, the samples were polished. This preparation work aimed to enable the better observation and analyzation of the microstructures of the rock samples. Figure 8 shows a photomicrograph of SJW sample production. Finally, the rock samples were observed using the Ultra55 field emission scanning electron microscope from Carl Zeiss, Oberkochen, Germany. Additionally, the composition of the samples was analyzed using energy dispersive X-ray spectroscopy (EDS) to understand their chemical compositions.

Through these meticulous operations and the use of advanced equipment, a deeper understanding of the microstructural changes in concrete microbars after 120 days of reaction can be achieved, providing valuable data support for research.

## 3. Results

### 3.1. Concrete Microbar Experiment

#### 3.1.1. Aggregate Alkali Activity

**Figure 9:**
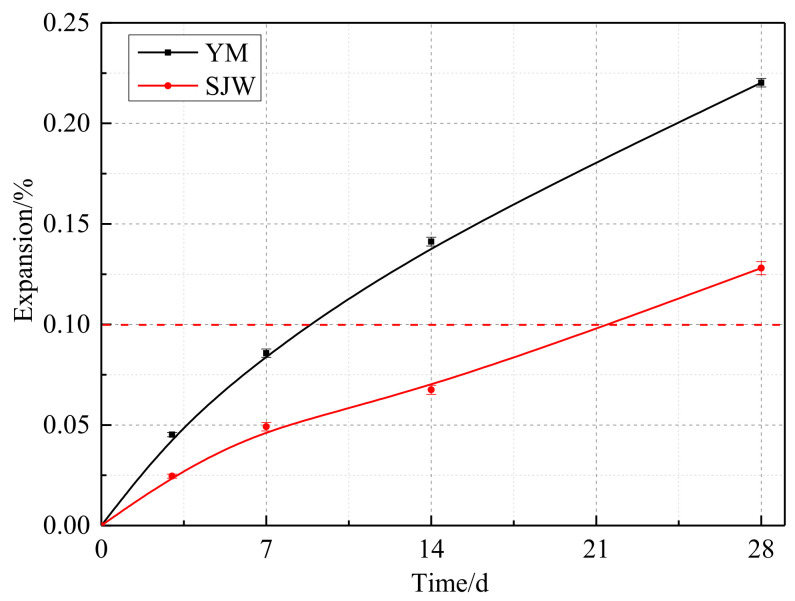
Expansion of the concrete microbars prepared according to RILEM AAR-5.

**Figure 10:**
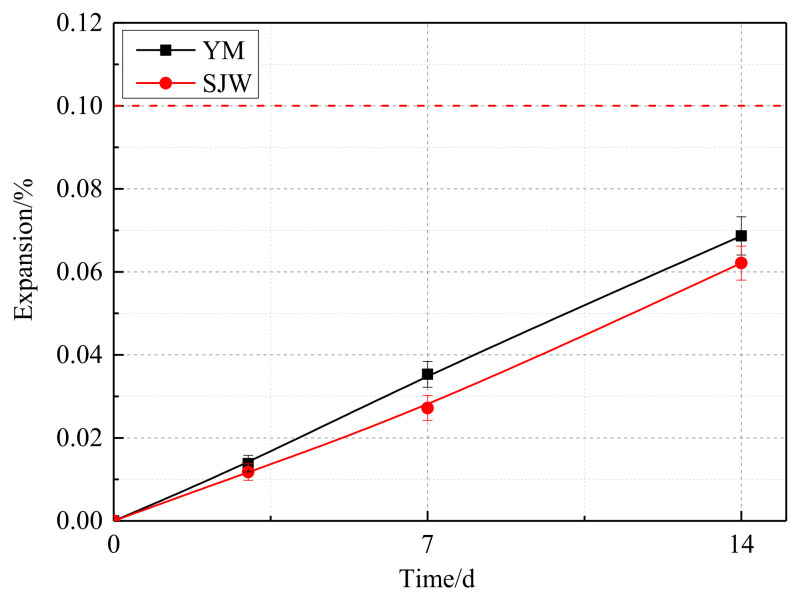
Expansion of the mortar bars prepared according to RILEM AAR-2.

In Figure 9, the expansion rates of SJW and YM at 28 days are 0.128% and 0.227%, respectively. According to RILEM AAR-5 standard, their expansion levels have exceeded the threshold of 0.1%, indicating the alkali–carbonate reaction activity of both SJW and YM aggregates. On the other hand, in Figure 10, the expansion rates of SJW and YM at 14 days are 0.062% and 0.682% (could be rounded down to 0.1%), respectively. According to RILEM AAR-2 standard, they do not exceed the critical value of 0.1%, indicating that SJW aggregates do not possess alkali–silica reaction activity. Therefore, combining the data from Figure 8 and Figure 9, it can be inferred that SJW and YM aggregates exhibit alkali–carbonate reaction activity but do not possess alkali–silica reaction activity.

#### 3.1.2. Effect of Cement Alkali Content on Concrete Microbar

**Figure 11:**
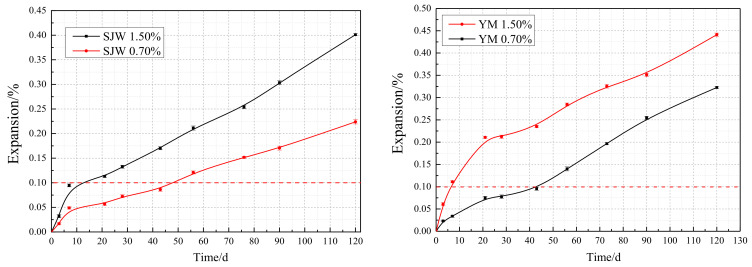
Expansion of SJW and YM when cement alkali content of 1.50% and 0.70%.

Figure 11 illustrates that when the cement alkali content is 1.50%, the expansion rates of concrete microbars made with SJW and YM rapidly increase, reaching 0.406% and 0.416%, respectively, after 120 days of reaction. As the cement alkali content decreases to 0.70%, the expansion rates of both types of rocks continue to show an increasing trend, with expansion rates reaching 0.22% and 0.32%, respectively. This result indicates that reducing the cement alkali content does not completely eliminate the expansion tendency of concrete microbars but rather exerts an inhibitory effect, slowing down the rate of expansion. It suggests that changes in cement alkali content have a direct and significant impact on the expansion performance of concrete microbars. Excessive or insufficient cement alkali content may lead to an increase in the expansion rate of concrete microbars, thereby affecting their long-term performance and durability. Therefore, when designing concrete mixtures, careful consideration should be given to the cement alkali content to optimize both the microstructure and macroscopic performance of concrete.

#### 3.1.3. Effect of Fly Ash on Concrete Microbars

**Figure 12:**
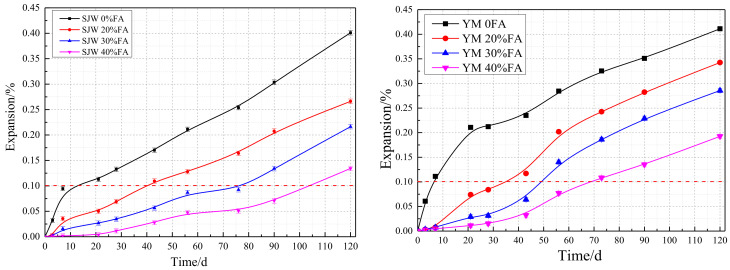
Expansion curves of concrete microbars with different fly ash contents under the same curing conditions and alkali content of 1.50% for SJW and YM.

In Figure 12, the comparative curve chart illustrates the influence of fly ash content at 0%, 20%, 30%, and 40% on the AAR expansion of concrete microbars made with SJW and YM, with a cement alkali content of 1.50%. It can be observed from the graph that without the addition of fly ash, the expansion rates of concrete microbars made with SJW and YM are relatively high. However, as the content of fly ash increases, there is a decreasing trend in the expansion rates. This result suggests that the addition of fly ash appears to suppress the alkali–aggregate reaction expansion of concrete microbars made with SJW and YM.

Based on the experimental results, the following conclusion can be drawn: With the increase in fly ash content, the AAR expansion of concrete microbars made from both types of rocks gradually decreases. This indicates that the addition of fly ash helps to mitigate the expansion of concrete microbars.

At 28 days, the expansion rates of concrete microbars without fly ash exceeded the failure criterion of 0.10%, reaching 0.13% and 0.21%. In contrast, the expansion rates of concrete microbars containing 20%, 30%, and 40% fly ash were 0.06%, 0.03%, and 0.02%, respectively, significantly lower than those without fly ash.

After 120 days of curing, the expansion rates of concrete microbars containing 20%, 30%, and 40% fly ash for both SJW and YM were all below the failure criterion of 0.10%, with values of 0.08%, 0.05%, and 0.03% for SJW and 0.04%, 0.06%, and 0.04% for YM.

Comparing the two types of rocks, although concrete microbars made with YM exhibited higher expansion rates at the same fly ash content, this difference may be attributed to the inherent properties of YM rock and requires further investigation.

Overall, the addition of fly ash significantly reduced the expansion rate of concrete microbars. The experimental data also reveals a notable trend: as the fly ash content increases, the extent of AAR expansion in concrete microbars made from SJW and YM significantly decreases. When the fly ash content is increased to 20%, 30%, and 40%, the expansion rate of SJW decreases by 48%, 74%, and 91%, respectively, while the expansion rate of YM decreases by 65%, 86%, and 95%, respectively. This series of data indicates that fly ash addition can significantly suppress AAR-induced expansion in the short term (28 days) and remains effective in the long term (120 days). After 120 days of curing, the expansion rates of SJW concrete microbars containing 20%, 30%, and 40% fly ash decreased by 34%, 47%, and 67%, respectively, while those of YM concrete microbars decreased by 8%, 18%, and 53%, respectively. This suggests that with increasing fly ash content, the long-term expansion performance of concrete microbars is further improved.

These results demonstrate that the addition of fly ash can effectively suppress AAR expansion in concrete microbars, enhancing their durability.

**Figure 13:**
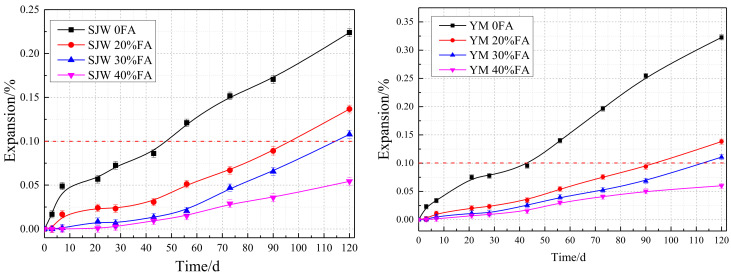
Expansion curves of concrete microbars with different fly ash contents under the same curing conditions and alkali content of 0.70% for SJW and YM.

Based on the experimental data in Figure 13, the following analysis can be conducted: When the cement alkali content is 0.70%, the expansion rates of SJW and YM concrete microbars without fly ash at 56 days are 0.12% and 0.14%, respectively, exceeding the failure criterion of 0.10%. This indicates that without fly ash, the concrete microbars exhibit some AAR expansion. After adding 30% and 40% fly ash, the expansion rates of SJW and YM concrete microbars at 56 days significantly decreased. The expansion rates of SJW decreased by 83% and 88%, respectively, while those of YM decreased by 72% and 78%, respectively. This suggests that the addition of fly ash effectively suppresses AAR expansion in concrete microbars. After 120 days of curing, although the expansion rates of SJW and YM concrete microbars with 30% and 40% fly ash increased slightly, they still remained below the 0.10% failure criterion. The expansion rates of SJW decreased by 48% and 76%, respectively, while those of YM decreased by 61% and 79%, respectively. This further confirms the effectiveness of fly ash addition in the long-term suppression of AAR expansion.

### 3.2. Micro-Structure Analysis

**Figure 14:**
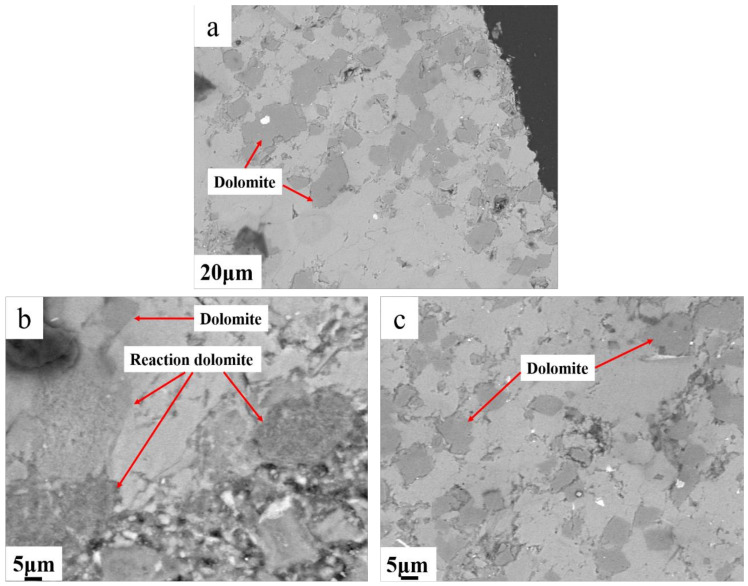
(**a**) BSEM images of dolomitic limestone SJW rock after reacting; (**b**)dedolomitization reactions within the region approximately 100 μm from the boundary; (**c**) no reactions were observed in the central area.

Concrete microbars with a cement alkali content of 1.50%, without fly ash, derived from SJW samples, were subjected to 120 days of curing at 80 °C. Detailed observations were conducted using BSEM. Figure 14 presents the BSEM images of SJW rock after reacting, revealing evident dedolomitization reactions within the region approximately 100 μm from the boundary, while no reactions were observed in the central area.

**Figure 15:**
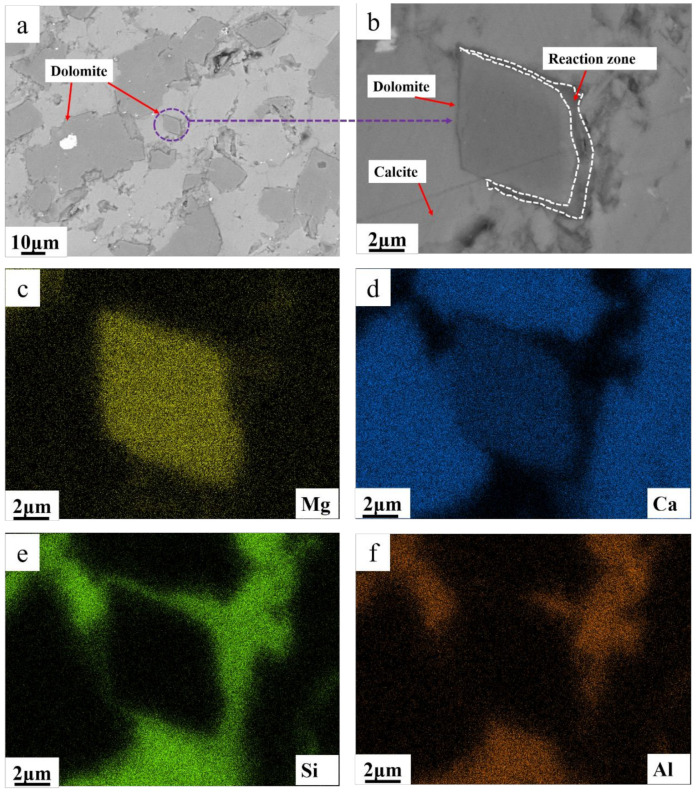
(**a**) BSEM images of SJW; (**b**) magnification of zone dolomite; (**c**) Mg map of magnified zone dolomite; (**d**) Ca map of magnified zone dolomite; (**e**) Si map of magnified zone dolomite; (**f**) Al map of magnified zone dolomite.

Through BSEM examination, the reactions were predominantly manifested in three forms: in Figure 15b, the dolomite region was magnified from Figure 15a, revealing distinct reaction rims around the dolomite grains. According to the EDS analysis results in Figure 15c–f, the surroundings of dolomite exhibited significant enrichment of clay minerals (primarily consisting of silicon and aluminum elements), with the presence of magnesium–silicon–aluminum phases detected within the reaction rims. Based on Li’s research findings [22], this indicates that upon dissolution of dolomite, magnesium ions react with clay minerals to form magnesium–silicon–aluminum phases. The formation of such phases impedes further erosion by alkaline substances, thereby resulting in relatively lower levels of erosion experienced by dolomite.

**Figure 16:**
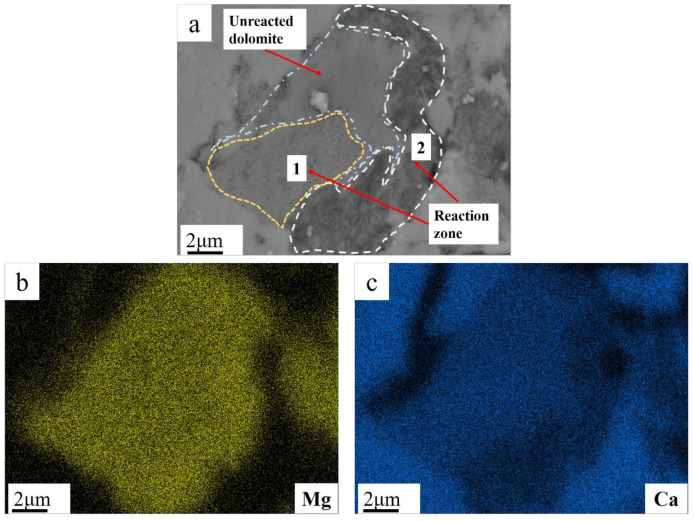
(**a**) BSEM images of SJW; (**b**) Mg map of magnified dolomite; (**c**) Ca map of magnified dolomite.

In Figure 16a, we observed the image of another dolomite, finding two distinct reaction regions. In reaction region 1, the surface of dolomite underwent a reaction, displaying a rough granular texture. Through EDS analysis, seen in Figure 16b,c, we identified the reaction products such as calcite and brucite, likely occurring in the initial stages of the reaction, forming a structure where reacted and unreacted dolomite are intergrown [23]. In contrast, reaction region 2 represents a fully reacted area, where the predominant reaction product is brucite. From these observations, we can conclude that the reaction of dolomite in cement is a complex process involving various forms of reactions and products.

**Figure 17:**
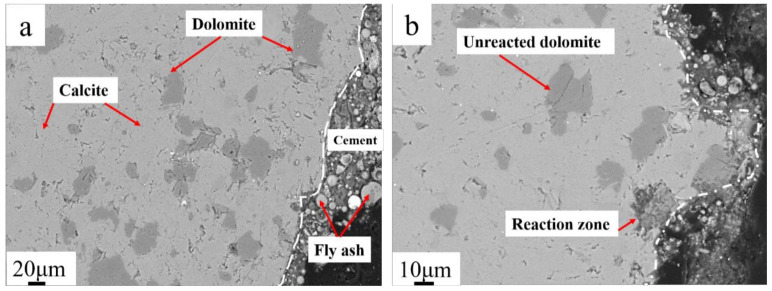
(**a**) BSEM image of SJW; (**b**) magnification of (**a**).

In Figure 17, the BSEM image after adding 40% fly ash is depicted. Figure 16b clearly reveals subtle variations at the edges of rock particles. It is noteworthy that in these edge regions, only a small amount of dolomite has undergone dedolomitization reaction. Upon further observation, it is found that in the areas closer to the interior, the effects of alkali attack are no longer present, and dolomite retains its original morphology. This finding is thought-provoking, as it suggests that the addition of fly ash not only inhibits the diffusion of alkali–aggregate reactions but also limits the extent of their impact.

**Figure 18:**
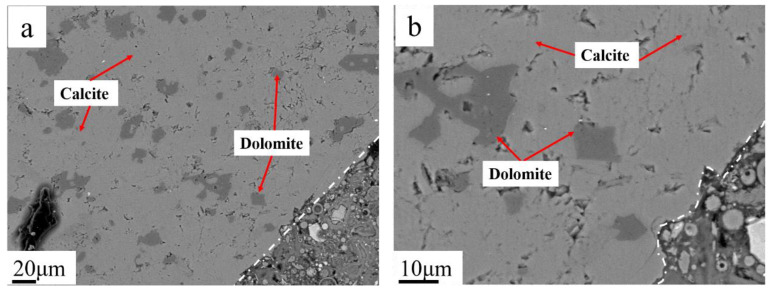
(**a**) BSEM image of SJW; (**b**) magnification of dolomite zone.

In Figure 18, BSEM images after reducing the cement alkali content to 0.70% are presented. In these images, dolomite near the edges exhibits intact and distinct boundaries, contrasting sharply with previous observations. In this region, no generation of black-and-white intergrown structures or black reaction rims is observed.

## 4. Discussion

A comparison of the swelling curves of the two rocks at different cement alkali contents revealed a sharp increase in the swelling rate at a cement alkali content of 1.50%. However, by reducing the alkali content to 0.70%, a significant reduction in the expansion rate was observed, although expansion was still observed. This suggests that simply lowering the cement alkali content is not an effective strategy for inhibiting the alkali carbonate reaction for aggregates with moderate to low reactivity. In practice, although cement alkali content is typically maintained below 1.50%, a cement alkali content of 0.70% is considered reasonable according to national standards. The causes of AAR include water, sufficient alkali, and active aggregate [2]. To inhibit ACR, the alkali content can be reduced. However, previous studies have shown that while reducing the cement alkali content can alleviate ACR, it cannot fully inhibit it [24]. There are two main reasons for this. Firstly, reducing the cement alkali content does not alter the pore structure of the slurry. Excessive harmful pores [25] in the slurry still facilitate the migration of OH^−^ into the aggregate. Secondly, cement hydration continues to produce Ca(OH)_2_, which also provides OH^−^ for ACR [26]. Further experiments revealed that even at a cement alkali content of 1.50% and a fly ash content of up to 40%, the alkali–carbonate reaction of medium- and low-reactive dolomitic limestone aggregates (SJW and YM) was not significantly suppressed. According to the alkali activity test criteria, when the cement alkali content was reduced to 0.70%, the late expansion exceeded 0.10%, which still indicated that the aggregates were potentially reactive and might pose a threat to the concrete structure. Data for fly ash contents between 20% and 40% showed that 20% and 30% fly ash contents failed to control expansion effectively. However, when the fly ash content was raised to 40%, the expansion curves leveled off and no upward trend was observed, implying the effectiveness of 40% fly ash content in suppressing the alkali–carbonate reaction expansion in both types of rocks. This is because the addition of fly ash refines the porosity, and at the same time, fly ash reacts with cement hydration Ca(OH)_2_ to generate C-S-H, which lowers the pH value inside the slurry and reduces the migration of OH^−^ to the inside of the aggregate, thus inhibiting the ACR [18,25].

In Qian’s study [27], it was found that the pH of the aggregate surface is higher than that of the interior, indicating a significant presence of OH^−^ on the aggregate surface, initiating the reaction from the surface. Li’s study [22] further demonstrated that the reaction products are dolomite and calcite. When the clay content is excessively high, Mg-Si-Al rings are formed, and the reaction gradually migrates towards the interior of the aggregate. This aligns with the experimental results. Using this criterion, the observation of the reaction area can reveal the effectiveness of adding fly ash. BSEM images further revealed the specific products of dedolomitization reactions. The study indicated that the main cause of alkali carbonate reaction is the dedolomitization reaction, with its products mainly including brucite and calcite. In cases where the clay content around dolomite is high, a reaction ring of Mg-Si-Al phase can form. In rock samples without fly ash, reaction products were mainly concentrated within 100 μm of the rock edge. However, with a 40% fly ash content, the reaction was significantly inhibited, with only a small amount of reaction found at the edge, possibly due to excessive external alkali content. When the cement alkali content decreased to 0.70%, no significant reaction was observed in the edge area. These results corroborate the concrete microbar expansion results, providing solid evidence for the study.

In summary, the addition of fly ash is an effective measure to improve the durability and long-term performance of concrete microbars, especially for concrete structures used in alkaline environments. However, it should be noted that the amount of fly ash added should be appropriate to ensure that the mechanical properties of concrete are not affected.

## 5. Conclusions

The aim of this study is to evaluate the ACR inhibition effect of medium- and low-activity dolomite limestone by observing the effect of fly ash. The concrete microcolumn method (RILEM AAR-5) was used as an evaluation criterion to experimentally seek the optimal combination of conditions for ACR inhibition in medium- and low-activity dolomite limestone. FESEM was utilized in the study to evaluate the ACR inhibition effect. Through the experimental results, this paper draws the following conclusions:

(1) Experimental results indicate that under a cement alkali content of 1.50%, it is challenging to accurately assess the inhibitory effect of fly ash on alkali carbonate reaction. When the alkali content is reduced to 0.70%, although the expansion rate decreases compared to the 1.50% alkali content condition, it still exceeds the threshold of 0.10% in the short term, indicating that reducing the alkali content may not effectively inhibit alkali carbonate reaction.
(2) In concrete microbars made with two different cement alkali contents and incorporating 20–40% fly ash, when the cement alkali content is 1.50%, none of the different proportions can inhibit alkali carbonate reaction. When the alkali content is reduced to below 0.70%, incorporating 20% and 30% fly ash still fails to suppress the reaction, while 40% fly ash achieves inhibitory effects.
(3) Through BSEM images, the products of dedolomitization reactions can be clearly observed. Analysis and observation of the reaction areas reveal that dedolomitization reactions mainly produce brucite, calcite, and Mg-Si-Al phases, with reactions primarily concentrated at the boundaries of the rocks. With the addition of fly ash, the reaction area significantly decreases, and when the cement alkali content decreases to 0.70%, successful control of the alkali carbonate reaction is achieved.

The results of these studies highlight that the incorporation of 40% fly ash in concrete structures containing moderately low reactive dolomitic limestone aggregates is effective in mitigating the alkali carbonate reaction, especially when the cement alkali content is controlled at 0.70%. Based on the experimental results, future work can include large-scale concrete exposure experiments for long-term observation. These experiments should include testing for flexural and compressive strength, as well as density, to verify the applicability of the working conditions.

## Figures and Tables

**Table 1:** Chemical compositions of cement from the Jiangnan Onoda Cement Company/wt%.

Component	LOI.	CaO	MgO	SiO_2_	Al_2_O_3_	Fe_2_O_3_	K_2_O	Na_2_O	SO_3_	Total
wt	2.81	64.00	2.35	19.43	4.73	2.96	0.47	0.26	2.58	99.59

**Table 2:** Chemical composition of FA/wt%.

Component	LOI.	CaO	MgO	SiO_2_	Al_2_O_3_	Fe_2_O_3_	K_2_O	Na_2_O	SO_3_	Total
wt	1.81	4.40	1.11	50.10	29.77	8.95	0.89	0.39	1.15	98.57

**Table 3:** Chemical compositions of rocks/wt%.

Simple	LOI.	SiO_2_	Fe_2_O_3_	Al_2_O_3_	CaO	MgO	Total
SJW	40.58	5.99	0.26	1.49	45.68	2.55	96.55
YM	37.19	11.18	1.03	2.80	42.26	2.48	96.94

## Data Availability

The raw data supporting the conclusions of this article will be made available by the authors on request.
